# 18. A case of anosmia

**DOI:** 10.1093/rap/rky033.010

**Published:** 2018-09-20

**Authors:** Hannah Zacharias, Malgorzata Magliano, Kieran Sandhu, Mark Weatherall

**Affiliations:** 1 Rheumatology, Stoke Manderville Hosital, Aylesbury, UNITED KINGDOM; 2 Neurology, Stoke Manderville Hosital, Aylesbury, UNITED KINGDOM

**Introduction:** Giant cell arteritis (GCA) is a common, well described medium and large-vessel vasculitis, which has a classical presentation occurring after the age of 50 with headache, scalp tenderness, polymyalgia and raised inflammatory response. Atypical presentations of systemic onset may lead to delay in diagnosis. Early diagnosis and treatment is essential to prevent often irreversible neurological complications. Although GCA predominantly affects the external carotid arteries and their branches it is a diffuse vascular disease that can affect arteries of any medium or large size. The level of vascular involvement can result in ischaemic manifestations such as visual loss, extraocular palsies, transient ischaemic attacks and infarcts have all been reported. Abnormalities of taste and smell are not typically found in giant cell arteritis. Olfactory involvement is rarely described, to date we have only identified two previous cases.

**Case description:** We describe the case of a 61 year old lady presenting a six month history of insidious onset hip and shoulder girdle pain and stiffness. She complained of malaise, weight loss 10lbs, night sweats and anorexia. Early on she noticed a loss of her sense of smell and after three months she had noticed alteration of taste specifically effecting red wine, apples, oranges and coffee. Appreciation of saltiness, bitterness and sweetness was unimpaired. Three months later she developed jaw claudication. She had no headaches, scalp tenderness or visual disturbance. She has longstanding hearing loss which runs in her family and asthma. She does not take any regular medications. There is no family history of autoimmune conditions. She is a non-smoker and takes no alcohol. On examination temporal and facial arteries were pulsatile and non-tender with normal peripheral pulses. Heart sounds were normal. She was normotensive with no arm discrepancy. Peripheral neurological exam was unremarkable, however smell was not formally assessed. There was no evidence of tongue necrosis which could contribute to taste loss. Eardrums normal with a decline in her hearing test at the higher frequencies. Investigations revealed a raised acute phase CRP 39 mg/L, ESR 44 mm/hr, normal FBC and slightly low albumin 32g/L. Histology of left temporal artery showed mild chronic inflammation of the arterial wall with mild to moderate intimal fibrosis, occasional giant cells and disruption of elastic lamina in keeping with giant cell arteritis (GCA). CT chest, abdomen and pelvis showed no evidence of malignancy or large vessel vasculitis. MRI showed normal brain appearance; normal pituitary fossa and no significant abnormality along the floor of the intracranial fossa, normal paranasal sinuses and nasal cavities. She was started on prednisolone for GCA with resolution of her symptoms other than anosmia and lack of taste. Over the next one year there had been a slight recovery in her sense of smell but she continues to experience a degree of dysosmia.

**Discussion:** On review of the neuroanatomy of the blood supply to the peripheral olfactory structures, the vasa nervorum of the olfactory bulb and nasal neuroepithelium derive their blood supply from the anterior and posterior ethmoid arteritis, which are branches of the ophthalmic branch of the internal carotid which is commonly affected in GCA. We feel that her symptoms were therefore a result of vasculitis and expect a degree of recovery with continued immunosuppression.

**Key Learning Points:** The awareness of these unusual symptoms should encourage physicians to pursue the diagnosis of GCA however it remains to be determined whether olfactory involvement is truly uncommon or simply underdiagnosed. As the number of older adults in the population increases, the incidence and burden of GCA will also increase, resulting in a need for heightened suspicion in both primary and secondary care.

**Disclosure: H. Zacharias:** None. **M. Magliano:** None. **K. Sandhu:** None. **M. Weatherall:** None.

